# Salivary Gland Extract of Kissing Bug, *Triatoma lecticularia*, Reduces the Severity of Intestinal Inflammation through the Modulation of the Local IL-6/IL-10 Axis

**DOI:** 10.1155/2018/1924393

**Published:** 2018-07-22

**Authors:** Helioswilton Sales-Campos, Jonatas da Silva Catarino, Guilherme Augusto Roza, Rafael Obata Trevisan, Luisa Menezes Silva, Juliana Reis Machado, Marcos Vinícius da Silva, Leonardo Euripedes Andrade-Silva, Virmondes Rodrigues-Júnior, Carlo José Freire de Oliveira

**Affiliations:** ^1^Instituto de Ciências Biológicas e Naturais, Universidade Federal do Triângulo Mineiro, Uberaba, MG, Brazil; ^2^Instituto de Patologia Tropical e Saúde Pública, Universidade Federal de Goiás, Goiânia, GO, Brazil; ^3^Departamento de Patologia Clínica, Universidade Federal do Triângulo Mineiro, Uberaba, MG, Brazil

## Abstract

Triatomines are known for their role as vectors of the causative agent of Chagas disease. The occurrence of an arsenal of molecules in their saliva is able to suppress vertebrate immune responses. Thus, it is reasonable to assume that the presence of molecules with therapeutic potential in their saliva is able to constrain inflammation in immune-mediated diseases. Thus, mice were exposed to dextran sulfate sodium (DSS) in drinking water uninterruptedly during 6 consecutive days and treated with *T. lecticularia* salivary gland extract (SGE) (3, 10, or 30 *μ*g) or vehicle (saline) (*n* = 6/group). At the highest dose (30 *μ*g), an improvement in clinical outcome and macroscopic aspects of the intestine were observed. This observation was followed by amelioration in histopathological aspects in the colon especially when the doses of 10 and 30 *μ*g were used. Regardless of the concentration used, treatment with *T. lecticularia* SGE significantly reduced the levels of the inflammatory cytokine IL-6 in the intestine. The production of the anti-inflammatory cytokine IL-10 was positively impacted by the concentrations of 3 and 30 *μ*g. Our results suggest that the presence of molecules in the *T. lecticularia* SGE is able to attenuate clinical outcome and colon shortening and improve intestinal architecture besides reducing the production of IL-6 and inducing a local production of IL-10 in the intestine.

## 1. Introduction

Triatomines are known for their role as natural vectors of *Trypanosoma cruzi*, the causative agent of Chagas disease, which is endemic in 21 Latin American countries and in the southern area of the United States [1]. Furthermore, because of migrations of infected people to nonendemic countries, Chagas disease can also be detected in Europe [2], Canada [3], Asia, and Oceania [4]. So far, 153 species of triatomines were identified and those belonging to the *Rhodnius*, *Triatoma*, and *Panstrongylus* genera are the major members of this group [5–7].

The species *Triatoma lecticularia* is predominantly observed in the United States and Mexico, being found in domestic and peridomestic areas [8]. This species seems to have a great capability to adapt to distinct environments ranging from rain forests to deserts, valleys, and laboratory conditions [8, 9]. Experimental data also suggested that *T. lecticularia* is an effective vector of *T. cruzi* to human populations [10]. Further, this species is able to adapt and replace other triatomines in the transmission of Chagas disease [9], besides being one of the most important species with potential to transmit Chagas disease in Texas, United States [11]. In nature, this triatomine is associated with terrestrial rodents [8]. Hence, this species shows great capability to transmit the causative agent of Chagas disease, to live and adapt under different environmental conditions, and to parasite distinct vertebrate hosts, including humans. These characteristics may be at least partly attributed to a variety of compounds in their saliva allowing them to properly feed, reproduce, and live.

After millions of years of coevolution with vertebrate hosts, triatomines, like other blood-feeding arthropods, developed in their saliva complex mechanisms to suppress vertebrate immune and hemostatic responses [12–14]. For this reason, studies have been focusing on the pharmacological potential of molecules in the saliva from different hematophagous arthropods to treat inflammatory/autoimmune conditions. Treatment using a salivary extract of *Phlebotomus papatasi* sandflies improved clinical signs, reduced the disease activity, and modulated dendritic cell (DC) and Th17 cell functions in an experimental model of rheumatoid arthritis [15]. Similarly, sialostatin L, a cysteine protease inhibitor identified in the salivary glands of *Ixodes scapularis* ticks, was able to reduce the severity of experimental autoimmune encephalomyelitis [16] and to prevent the occurrence of asthma in rodents [17]. In addition, the administration of *Aedes aegypti* saliva presented a beneficial effect on the clinical outcome of intestinal inflammation induced by dextran sulfate sodium (DSS) [18]. The amelioration of the clinical parameters was followed by an inhibition in the production of IFN-*γ*, TNF-*α*, IL-1*β*, and IL-5 together with a reduction in the inflammatory area locally in the intestine [18].

Our group has previously demonstrated the *in vitro* effect of *T. lecticularia* salivary gland extract (SGE) on DCs stimulated with LPS [14]. The presence of *T. lecticularia* SGE in DC cultures reduced the production of TNF-*α*, IL-6, and IL-12 besides inducing the production of IL-10 in a concentration-dependent manner with no negative impact on cell viability [14]. However, to the best of our knowledge, the therapeutic role of salivary molecules of triatomines in inflammatory diseases has never been addressed. Thus, the unexplored pharmacological potential of triatomine saliva, especially *T. lecticularia*, prompted us to investigate the therapeutic potential of the saliva of this species in the treatment of experimentally induced intestinal inflammation.

## 2. Methods

### 2.1. *T. lecticularia* Salivary Gland Extract (SGE)

*T. lecticularia* males and females were reared in the insect facility at the Institute of Biological and Natural Sciences, Federal University of Triângulo Mineiro. Adults of *T. lecticularia* were cleaned in 70% ethanol and water to be dissected in saline, and their salivary glands were transferred to a tube containing cold saline. Every three pairs of glands (representing three insects) were added to 10 *μ*l of saline. Subsequently, this material was grinded using sterile needles and centrifuged at 10,000*g* to remove insoluble material. The supernatant (SGE) was removed, and the protein concentration was determined by a NanoDrop 2000 spectrophotometer (Thermo Fisher Scientific, Wilmington, DE, USA). Aliquots were stored at −80°C until use. All specimens used in this study were classified and identified as previously described [19]. The insects used in this experiment were from F2 generation.

### 2.2. Mice, Colitis Induction, and Treatment

Male C57BL/6 mice aged 6–8 weeks were maintained on a 12/12 h light/dark cycle with water and food *ad libitum* in the animal facility at the Federal University of Triângulo Mineiro (UFTM), Brazil. Mice were divided into groups of 6 animals each as follows: *naïve* animals (control group), healthy mice without intestinal inflammation treated with the highest concentration of *T. lecticularia* SGE (control + 30 *μ*g), mice with intestinal inflammation induced by dextran sulfate sodium (DSS, MP Biomedicals, Illkirch, France; molecular weight: 36,000–50,000) treated with sterile saline as a vehicle (DSS + saline), and mice with intestinal inflammation treated with *T. lecticularia* SGE in three different concentrations (DSS + 3, 10, or 30 *μ*g). For colitis induction, mice were exposed to 3% (*w*/*v*) DSS uninterruptedly in drinking water during 6 consecutive days. Treatment was performed by intraperitoneal (i.p.) route from the beginning of the exposure to DSS (day 0) until the day before euthanasia (day 5). Thus, each mouse was treated with *T. lecticularia* SGE in 0.1 ml sterile saline or saline only. All experimental procedures were reviewed and approved by the Institutional Animal Care and Use Committee of UFTM (protocol number 371) and performed according to the criteria outlined by the Brazilian Society for Laboratory Animal Science (SBCAL).

### 2.3. Clinical Assessment

Body weight variation and clinical signs of disease were evaluated daily to obtain a clinical disease score for every mouse. Each clinical sign presented by the mice corresponded to one point, and the sum of points for each mouse outlined a clinical score. Clinical scores were determined as previously described [18].

### 2.4. Euthanasia and Sample Collection

Mice were euthanized on day 6, and the colon was removed for further analysis. A picture from each intestine was taken, and the intestinal length was analyzed using ImageJ (National Institutes of Health (NIH), Bethesda, MD, United States). Colon samples were divided into smaller sections that were immediately frozen in liquid nitrogen for quantification of nitric oxide (NO) activity. Some sections were immersed into phosphate-buffered saline (PBS)/10% formaldehyde for paraffin embedding. For cytokine quantification by enzyme-linked immunosorbent assay (ELISA), samples were collected in PBS containing a protease inhibitor (Complete®, Roche Pharmaceuticals, Mannheim, Germany).

### 2.5. Nitric Oxide (NO)

NO production was assessed based on the levels of nitrite accumulation in intestinal homogenates using Griess reaction as previously described [20] with some modifications [21]. The readings were performed at 540 nm in a 96-well plate reader (Perkin Elmer Cetus, San Jose, CA, USA). The total amount of nitrite in the intestinal samples was calculated based on the absorbance of the serial dilution of sodium nitrite standard curve. Then, results were normalized to the dry weight of each intestinal section and expressed as micrograms per milliliter per gram of tissue (*μ*g/ml/g).

### 2.6. Cytokine Quantification by ELISA

The levels of IL-10, IFN-*γ*, TNF-*α*, IL-10, and IL-6 were determined in tissue homogenates by ELISA following the manufacturer's instructions (BD Biosciences, San Jose, CA, USA). Results were normalized to the dry weight of each intestinal section and expressed as nanograms per milliliter per gram of tissue (ng/ml/g).

### 2.7. Histopathological Analysis

To assess the impact of exposure to DSS and treatment using *T. lecticularia* SGE on intestinal architecture, tissue sections were washed with PBS, fixed in 10% buffered formalin for 24 h, and then processed for paraffin embedding followed by microtome sectioning. 5 *μ*m sections were obtained and stained with hematoxylin and eosin (H&E). The following areas were analyzed to verify disturbances induced by DSS and the impact of treatment using the triatomine saliva: mucosa, submucosa, muscle layers, and serosa. Additionally, intestinal sections were also assessed for the presence of edema, inflammatory infiltrate, and epithelial abnormalities.

Morphometry was performed using Image-Pro Insight (Media Cybernetics). The inflammatory infiltrate was determined based on the damaged area containing inflammatory infiltrate divided by the total area of tissue visualized in the acquired image and expressed as a percentage (%). A trained pathologist who was blinded to the treatment performed the histopathological analysis.

### 2.8. Data Analysis and Statistics

Normal distribution and homogeneous variance were tested for all of the variables. When the distribution was considered normal and the variance was homogeneous, parametric tests were used: unpaired Student's *t*-test or one-way ANOVA followed by Tukey's posttest. In cases of non-Gaussian distribution of data, the Mann–Whitney or Kruskal-Wallis tests following nonparametric tests followed by Dunn's test were used. The differences were considered significant when *p* < 0.05 (5%). The results were expressed as mean ± SD. Statistical analysis was performed using GraphPad Prism 6.0 (La Jolla, CA, USA).

## 3. Results

### 3.1. *T. lecticularia* SGE Improves Clinical Outcome in DSS-Induced Colitis

**Figure 1:**
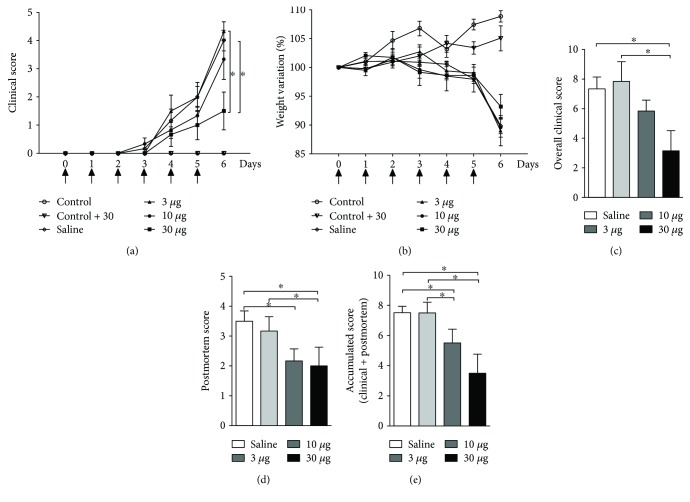
Treatment with *T. lecticularia* SGE improves clinical outcome in mice exposed to DSS. Intestinal inflammation was induced by free consumption of drinking water containing 3% DSS for 6 consecutive days. Mice were treated i.p. with 100 *μ*l of saline or *T. lecticularia* SGE (3, 10, or 30 *μ*g/animal/day) as depicted in the figure (arrows). The clinical disease score (a), weight variation (b), overall clinical score (c), postmortem score (d), and accumulated score (e) were recorded during the disease development or at the day of euthanasia. Results are representative of two independent experiments (*n* = 6 mice/group). ^∗^*p* < 0.05.

To evaluate whether the *T. lecticularia* salivary molecules would be able to improve clinical signs of colitis, mice were i.p. treated with 3, 10, or 30 *μ*g of the preparation described in Methods. These doses were chosen based on previous results from our lab (data not shown). Among the different doses tested, the highest one (30 *μ*g) led to the amelioration in clinical outcome and overall clinical score, especially when compared to mice treated with 3 *μ*g or saline (Figures 1(a) and 1(c), resp.). The beneficial effect of the highest concentration of *T. lecticularia* SGE (30 and 10 *μ*g) is highlighted by the reduction in postmortem and accumulated scores when compared to the results observed in mice treated with 3 *μ*g or saline (Figures 1(d) and 1(e), resp.). Treatment with triatomine SGE seemed not to impact weight loss (Figure 1(b)). Taken together, our results suggest that *T. lecticularia* SGE is able to improve colitis outcome, reducing the disease severity especially when the highest concentration is used.

### 3.2. Treatment with *T. lecticularia* SGE Improves Intestinal Shortening and Histopathological Alterations in Mice Exposed to DSS

**Figure 2:**
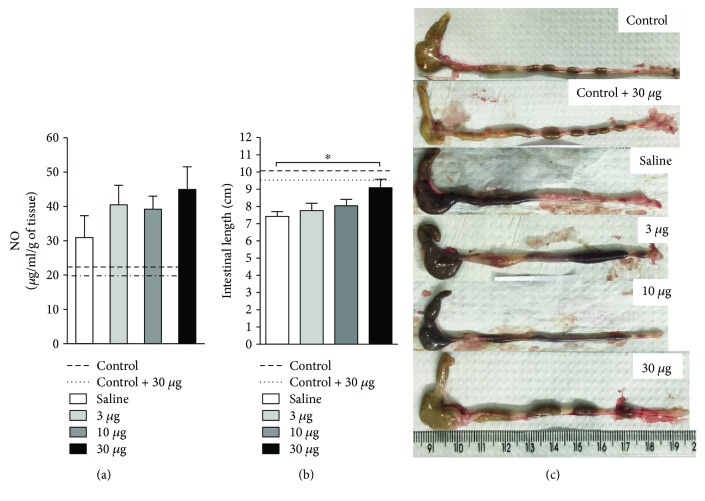
*T. lecticularia* SGE improves intestinal shortening in mice exposed to DSS. Intestinal inflammation was induced by free consumption of drinking water containing 3% DSS for 6 consecutive days. Mice were treated i.p. with 100 *μ*l of saline or *T. lecticularia* SGE (3, 10, or 30 *μ*g/animal/day). Dashed lines: healthy mice without treatment and not exposed to DSS (control); dotted lines: healthy mice not exposed to DSS and treated with *T. lecticularia* SGE (30 *μ*g). The NO production (a) was assessed as described in Methods. Results were normalized to the dry weight of each intestinal section and expressed as micrograms per milliliter per gram of tissue (*μ*g/ml/g). The intestinal length (b, c) was recorded at the day of euthanasia. Results are representative of two independent experiments (*n* = 6 mice/group). ^∗^*p* < 0.05.

As we have observed the positive impact of *T. lecticularia* SGE on colitis outcome, we addressed how this effect could be related to the amelioration in intestinal architecture and local inflammation. Regardless of the concentration used or the nature of treatment (saliva or vehicle), no alterations were observed for the production of NO (Figure 2(a)). Interestingly, treatment with the highest dose (30 *μ*g) improved intestinal shortening when compared to mice treated with the other concentrations (3 or 10 *μ*g) or saline alone (Figures 2(b) and 2(c)).

**Figure 3:**
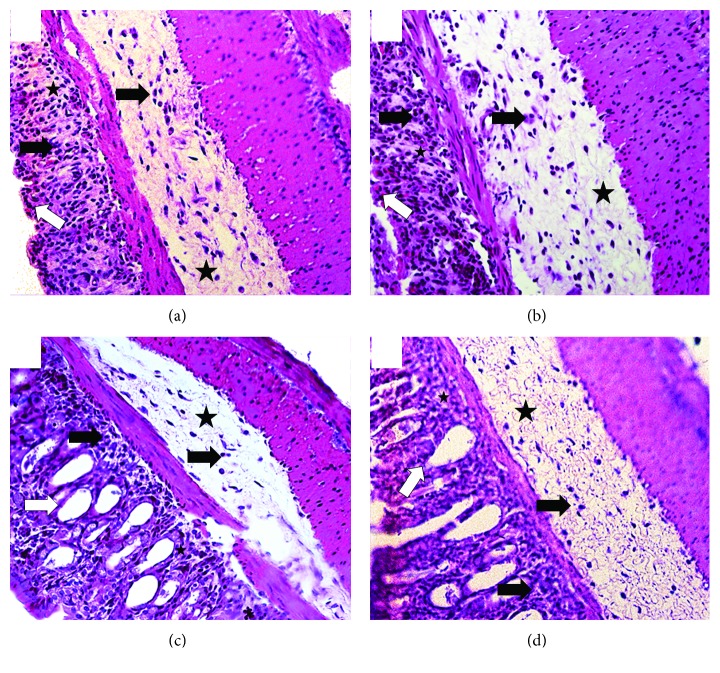
*T. lecticularia* SGE improves histopathological alterations in mice exposed to DSS. Intestinal inflammation was induced by free consumption of drinking water containing 3% DSS for 6 consecutive days. Mice were treated i.p. with 100 *μ*l of saline or *T. lecticularia* SGE (3, 10, or 30 *μ*g/animal/day). (a) Mice exposed to DSS: lamina propria with moderate edema (asterisk) and intense mononuclear cell infiltration (black arrow) and submucosa with moderate mononuclear infiltrate (black arrow) and intense edema (asterisk). Severely damaged crypts (white arrow); (b) mice exposed to DSS and treated with *T. lecticularia* SGE (3 *μ*g/animal/day): lamina propria with moderate edema (asterisk) and mononuclear cell infiltration (black arrow) and submucosa with moderate mononuclear infiltrate (black arrow) and moderate edema (asterisk). Severely damaged crypts (white arrow); (c) mice exposed to DSS and treated with *T. lecticularia* SGE (10 *μ*g/animal/day): lamina propria with discrete edema (asterisk) and mononuclear cell infiltration (black arrow) and submucosa with discrete mononuclear infiltrate (black arrow) and edema (asterisk). Dilated crypts (white arrow); (d) mice exposed to DSS and treated with *T. lecticularia* SGE (30 *μ*g/animal/day): lamina propria with discrete edema (asterisk) and mononuclear cell infiltration (black arrow) and submucosa with moderate mononuclear infiltrate (black arrow) and edema (asterisk). Partly damaged or dilated crypts (white arrow). Results are representative of two independent experiments (*n* = 6 mice/group).

Next, we aimed to investigate whether the preservation of intestinal length was associated with amelioration in local architecture of the organ. Mice treated with the two highest doses (10 and 30 *μ*g) had discrete lamina propria swelling and mononuclear infiltrate in the mucosal and submucosal layers, especially when the dose of 10 *μ*g was used (Table 1). Additionally, treatment at the highest doses (10 and 30 *μ*g) was able to reduce the severity of the damage (Figures 3(c) and 3(d), resp.) in intestinal crypts (Table 1). On the other hand, mice treated with the lowest concentration (3 *μ*g) (Figure 3(b)) or vehicle (saline) (Figure 3(a)) had their crypts severely compromised (Table 1). Altogether, these results suggest a positive impact of *T. lecticularia* SGE on preventing intestinal shortening besides reducing the severity of histopathological alterations in mice exposed to DSS.

### 3.3. *T. lecticularia* SGE Modulates Key Cytokines Related to Colitis Outcome

**Figure 4:**
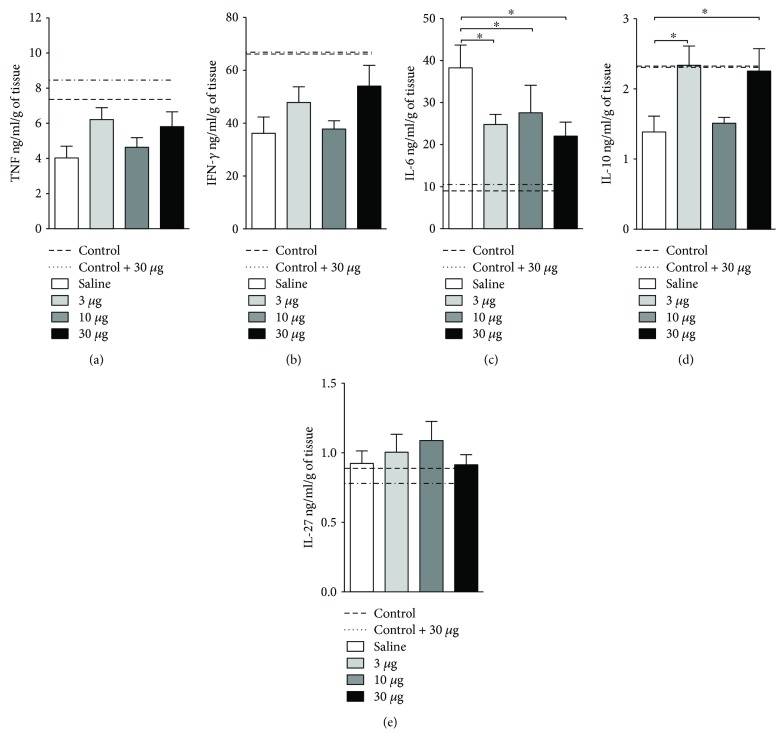
*T. lecticularia* SGE improved intestinal shortening in mice exposed to DSS. Intestinal inflammation was induced by free consumption of drinking water containing 3% DSS for 6 consecutive days. Mice were treated i.p. with 100 *μ*l of saline or *T. lecticularia* SGE (3, 10, or 30 *μ*g/animal/day). Dashed lines: healthy mice without treatment and not exposed to DSS (control); dotted lines: healthy mice not exposed to DSS and treated with *T. lecticularia* SGE (30 *μ*g). The concentration of TNF-*α* (a), IFN-*γ* (b), IL-6 (c), IL-10 (d), and IL-27 (e) was detected in intestinal sections collected with protease inhibitors. Results were normalized to the dry weight of each intestinal section and expressed as nanograms per milliliter per gram of tissue (ng/ml/g). Results are representative of two independent experiments (*n* = 6 mice/group). ^∗^*p* < 0.05.

Next, we aimed to elucidate if the amelioration observed in the clinical outcome and intestinal architecture was related to the modulation of cytokines locally in the intestine. Regardless of the concentration of *T. lecticularia* SGE used, a significant reduction in the production of the colitogenic cytokine IL-6 was observed in the intestine when compared to mice treated with saline only (Figure 4(c)). Additionally, treatment using 3 or 30 *μ*g of SGE augmented the levels of IL-10 in the intestine when compared to mice treated with 10 *μ*g or vehicle only (Figure 4(d)). No differences were observed for the production of TNF-*α* (Figure 4(a)), IFN-*γ* (Figure 4(b)), and IL-27 (Figure 4(e)). These results pointed to the beneficial role of the treatment with *T. lecticularia* saliva in the modulation of key cytokines related to colitis worsening.

## 4. Discussion

Triatomines, such as *T. lecticularia*, are widely known by their role as vectors of Chagas disease. However, the pharmacological potential of their salivary compounds has never been addressed before. Here, we report for the first time the therapeutic effect of *T. lecticularia* SGE on the outcome of intestinal inflammation induced by DSS. The amelioration of clinical and macroscopic aspects seemed to be associated with a reduction in clinical scores, preservation of mucosal architecture, diminishment of local inflammatory infiltrate, and modulation of key cytokines.

The direct correlation between clinical improvement and low rate of side effects represents one of the primary targets in the development of new approaches to treat inflammatory bowel disease (IBD). It is well known that clinical score and its correlated indexes are the main parameters to estimate the level of intestinal inflammation and successfulness of the treatment. However, other aspects, such as colon lengthening, may also be used as an indirect index in this scenario. In the present study, treatment using the highest concentration of *T. lecticularia* saliva besides improving clinical outcome and macroscopic parameters in the intestine also had a positive impact on reducing intestinal shortening.

Immune-mediated diseases, like IBD, are multifactorial disorders where environmental triggers, genetic predisposition, and immune disturbances contribute to disease pathogenesis [22]. In this scenario, current therapies are aimed at modulating the immune system to reduce the severity of inflammation and prolonging periods of remission. Within the immune aspects that dictate the pathogenesis of IBD in the intestinal mucosa, the imbalance between effector and regulatory immune responses plays a central role in disease onset and outcome [23, 24]. This immune disturbance triggers both innate and adaptive immune responses that in turn produce a wide range of cytokines [25], thus leading to uncontrolled inflammation. For this reason, one of the targets of therapeutic approaches to treat IBD consists in the neutralization of inflammatory players like cytokines. Immunotherapies using neutralizing antibodies targeting TNF-*α* [26], IL-12/IL-23 subunit p40 [27], or integrins [27] have been successfully used to treat moderate-to-severe Crohn's disease (CD). To note, IBD is especially composed of two different entities CD and ulcerative colitis (UC) [28]. However, a considerable number of patients are refractory or intolerant to these therapies, particularly those targeting TNF-*α* [29]. This scenario highlights the necessity for developing new therapeutic approaches to treat IBD. In our study, *T. lecticularia* saliva was effective in the reduction of the colitogenic cytokine IL-6. This molecule is a key modulator in inflammatory response and presents deleterious effects on the pathogenesis of IBD such as inhibition of apoptosis in mucosal T cells and/or favoring the differentiation of Th17 cells [30]. For this reason, different studies have been targeting IL-6 to treat IBD. A clinical trial conducted with 247 patients induced clinical response and remission, after treatment with anti-IL-6, in refractory patients with moderate-to-severe CD following failure of anti-TNF therapy [31]. A different clinical trial conducted with a smaller number of individuals (*n* = 36) with active CD also showed the positive effects of treatment targeting IL-6 [32]. Though we have not explored the effects of *T. lecticularia* saliva on the apoptosis of mucosal T cells nor its effects on the differentiation of T cells, like Th17 lymphocytes, we cannot underestimate its importance in this context. Our group has shown the ability of *T. lecticularia* SGE to reduce the differentiation and production of IL-6 in dendritic cells [14]. Because of the role of these cells in antigen presentation and cell activation or differentiation, the contribution of such modulation to disease amelioration cannot be ruled out.

The reestablishment of immune balance represents a great challenge in the treatment of immune-mediated diseases, like IBD. In our study, treatment with *T. lecticularia* SGE reduced the production of the inflammatory cytokine IL-6 besides improving the production of IL-10 locally in the intestine. IL-10 presents great anti-inflammatory activity, and mutations in *IL-10* and its receptor (*IL-10R*) were associated with early onset and severity of IBD [33–35]. IL-10 plays a crucial role in the maintenance and regulation of mucosal immune tolerance and in the anti-inflammatory function of macrophages in the intestine [36]. In mice lacking the IL-10R, intestinal inflammation and macrophage dysfunction begin during the third week of life [37]. The role of macrophages in this context is highlighted when these cells are depleted leading to the protection from the development of colitis even in IL-10R-deficient mice [37]. Despite the importance of macrophages in this context, we have only addressed the effects of salivary molecules on the development of DCs. The anti-inflammatory activity of macrophages in the intestinal mucosa seems to be dependent on the IL-10/p38*α* MAPK pathway [38]. The p38 MAPK pathway was formerly involved in the regulation of proinflammatory cytokines by myeloid cells [39]. However, the p38 MAPK cascade seems to have an ambiguous role in inflammation and can present pro- or anti-inflammatory effects [40], depending on the context. Additionally, the production of IL-10 seems also to be negatively influenced by the inhibition of p38*α*, one of the four isoforms of the p38 MAPK pathway [38]. These results suggest the importance of IL-10 to the maintenance of intestinal homeostasis besides its crucial role in IBD onset and worsening. Furthermore, due to the complexity of targeting one single cytokine to constrain inflammation in IBD, a reduction in IL-6 coupled with higher levels of IL-10 related to the treatment using *T. lecticularia* SGE may represent a promising approach to treat immune-mediated diseases. However, the mechanisms concerning the impact of *T. lecticularia* SGE on specific aspects of the immune system as well as the molecules responsible for the effects observed still need to be clarified.

## 5. Conclusion

Overall, our data suggested the presence of molecules with pharmacologic potential in *T. lecticularia* SGE. Treatment with this triatomine SGE besides improving clinical outcome and reducing the severity of damage in the intestinal epithelia also reestablished the immune balance in the intestine. Nonetheless, further studies must be performed in order to elucidate the molecules in *T. lecticularia* SGE responsible for the effects observed.

## Figures and Tables

**Table 1:** Histopathological alterations in mice exposed to DSS and treated or not with *T. lecticularia* SGE.

Groups	Control	Control + 30 *μ*g	Saline	3 *μ*g	10 *μ*g	30 *μ*g
Lamina propria swelling	Absent	Absent	Moderate	Moderate	Discrete	Discrete
Submucosa swelling	Absent	Discrete	Intense	Moderate	Moderate	Moderate
Mononuclear infiltrate in the mucosal layer	Absent	Absent	Intense	Moderate	Discrete	Moderate
Mononuclear infiltrate in the submucosal layer	Absent	Absent	Moderate	Moderate	Discrete	Moderate
Crypts	Preserved	Preserved	Severely damaged	Severely damaged	Dilated	Partly damaged

## Data Availability

Data are available upon request.
